# Functional analysis of three new alpha-thalassemia deletions involving MCS-R2 reveals the presence of an additional enhancer element in the 5’ boundary region

**DOI:** 10.1371/journal.pgen.1010727

**Published:** 2023-05-22

**Authors:** Serena Capasso, Giovanna Cardiero, Gennaro Musollino, Romeo Prezioso, Rosario Testa, Sabrina Dembech, Giulio Piluso, Vincenzo Nigro, F. Anna Digilio, Giuseppina Lacerra

**Affiliations:** 1 Institute of Genetics and Biophysics "Adriano Buzzati Traverso" (IGB-ABT), National Research Council (CNR), Naples, Italy; 2 A.O.U. Policlinico Rodolico-San Marco, University of Catania, Catania, Italy; 3 Central analysis laboratory, Azienda Ospedaliero-Universitaria, Ospedali Riuniti, Foggia, Italy; 4 Department of Precision Medicine, University of Campania L. Vanvitelli, Naples, Italy; 5 Telethon Institute of Genetics and Medicine (TIGEM), Pozzuoli, Italy; 6 Research Institute on Terrestrial Ecosystems (IRET-CNR), National Research Council (CNR), Naples, Italy; University of Pennsylvania, UNITED STATES

## Abstract

We report three novel deletions involving the Multispecies Conserved Sequences (MCS) R2, also known as the Major Regulative Element (MRE), in patients showing the α-thalassemia phenotype. The three new rearrangements showed peculiar positions of the breakpoints. 1) The (αα)ES is a telomeric 110 kb deletion ending inside the MCS-R3 element. 2) The (αα)FG, 984 bp-long, ends 51 bp upstream to MCS-R2; both are associated with a severe α-thalassemia phenotype. 3) The (αα)CT, 5058 bp-long starts at position +93 of MCS-R2 and is the only one associated to a mild α-thalassemia phenotype. To understand the specific role of different segments of the MCS-R2 element and of its boundary regions we carried out transcriptional and expression analysis. Transcriptional analysis of patients’ reticulocytes showed that (αα)ES was unable to produce α2-globin mRNA, while a high level of expression of the α2-globin genes (56%) was detected in (αα)CT deletion, characterized by the presence of the first 93 bp of MCS-R2. Expression analysis of constructs containing breakpoints and boundary regions of the deletions (αα)CT and (αα)FG, showed comparable activity both for MCS-R2 and the boundary region (-682/-8). Considering that the (αα)CT deletion, almost entirely removing MCS-R2, has a less severe phenotype than the (αα)FG α^0^thalassemia deletion, removing both MCS-R2 almost entirely and an upstream 679 bp, we infer for the first time that an enhancer element must exist in this region that helps to increase the expression of the α-globin genes. The genotype-phenotype relationship of other previously published MCS-R2 deletions strengthened our hypothesis.

## Author summary

The regulation of gene expression occurs through the physical interaction between the promoters, regions preceding the genes, and the enhancer, short regions placed even at a considerable distance, with a mechanism defined as long-range regulation.

Globin genes are a good model of gene regulation, due to the easy access to the target tissue (blood) and to the analyses that can be performed on it. In a study of patients with alpha-thalassemia, we identified three new deletions involving different parts of the enhancer named Major Regulative Element MCS-R2 associated with severe phenotype, and one of them, with mild. These deletions have given us the opportunity to gain insight into long-range regulation *in vivo*, using transcriptional analysis on reticulocytes from patients to analyze the role of individual regulatory elements.

Our results highlight for the first time a functional enhancer role associated to the boundary MCS-R2 regions and shed light on aspects related to long-range regulation. The data could be useful to better understand the pathophysiology of deletions involving the enhancer MCS-R2 and also for constructing expression vectors for gene therapy and for identifying drug target sequences to modulate gene expression.

## Introduction

The analysis of the globin genes expression and erythropoiesis, in the last 50 years, has been a model for understanding the mechanisms underlying the mammalian gene regulation during differentiation and development [1,2]. A significant contribution in identifying the regulatory element for the understanding of the long-range regulation has been made through the analysis of α-globin genes cluster.

**Fig 1:**
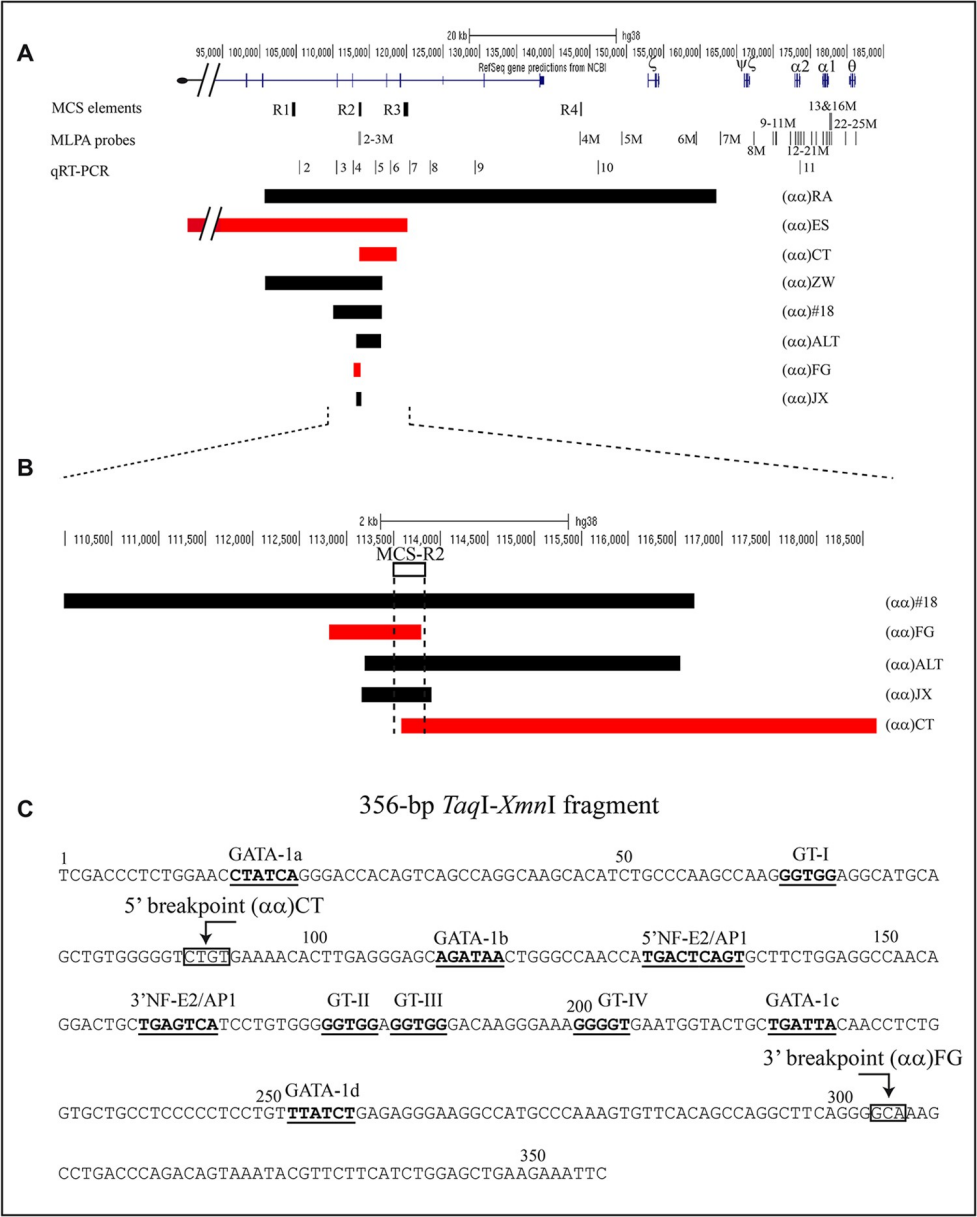
Scheme of deletions involving MCS-R2 and its structure. (A) The short arm of the chromosome 16 is reported and the genes of the α-globin gene cluster are indicated with Greek symbols. The black boxes are the four MCS elements. 2M to 25M are the MLPA probes; 2 to 11 the quantitative Real Time PCR (qRT-PCR) fragments amplified to localize the position of the deletions (see Methods). The relative position and the extension of the new (in red) (αα)ES, (αα)CT and (αα)FG and the known (in black) (αα)RA [14], (αα)ZW [15], (αα)#18 [16], (αα)ALT [17,18] and (αα)JX [19,20] deletions are reported on the chromosome scheme. (B) Zoom of the MCS-R2 element region with the relative position and the extension of the new (αα)FG and (αα)CT deletions and the known (αα)#18, (αα)ALT and (αα)JX deletions. (C) 356-bp TaqI-XmnI MCS-R2 element is reported [5,6]. It contains four consensus protein-binding site for the transcription factor GATA-1, two for the NF-E2/AP-1 and four GT sites. The (αα)CT 5’ breakpoint from position +89/+92 (CTGT is in the box), and the (αα)FG 3’ breakpoint from position +303/+305 (GCA is in the box) is reported.

The α-globin gene cluster, located on the short arm of the chromosome 16, includes in the order 5’-3’ the embryonic ζ-gene and the two duplicated fetal/adult α-globin genes (Fig 1A) [3].

The absence or reduction of the α-globin chains synthesis, caused the hereditary anemia called α-thalassemia. The phenotype of the heterozygotes is characterized by microcytosis and hypochromia. Heterozygotes may be affected by mild (α^+^thalassemia) or severe hematological alterations (α^0^thalassemia). Indeed, the alterations are related to the type and number of inactivated α-globin genes [3].

Four regulatory elements, called Multispecies Conserved Sequences (MCS) (from MCS-R1 to MCS-R4), also known as HS-48, HS-40, HS-33 and HS-10 (Fig 1A), and corresponding to the erythroid-specific DNase I Hypersensitive Sites (HSs) are present upstream of the cluster [4].

Studies of stable cell lines and transgenic mice, and on the humanized mouse model, provided evidence that the MCS-R2 element, about 350 bp-long, has the role of the α-globin Major Regulatory Element (α-MRE-R2) [5]. Analysis of the MCS-R2 element with genomic footprinting indicated the presence of binding sites for transcriptional factors such as GATA-1, NF2-E2/AP-1, CAC/GT (Fig 1C) [6–8].

Quantitative chromosome conformation capture (q3C) highlighted a looping model in which multiprotein complexes at the MCS-R2 and the gene promoters collide, and their interactions become stabilized, in erythroid cells [9]. The relative contributions of the α-globin enhancers to globin gene expression in the human has been suggested to be ~ 10% (MCS-R1), 90% (MCS-R2), 2% to 3% (MCS-R3), and 2% to 3% (MCS-R4) [5,10].

The identification of the deletions of the natural α-globin genes cluster and the analysis of their perturbed expression allowed to define the role of the MCS elements in the normal erythroid environment and to understand the molecular basis of human defects [1–3]. To date, 24 deletions upstream of the α-globin cluster -leaving the structural α-globin genes intact- have been described. Out of them 10 are telomeric and at least 100 kb long, whereas 14 remove regions ranging from 60 to few kb [11,12].

In this study, we report the molecular characterization of three new deletions involving the MCS-R2 element [13].

One deletion, associated with the α^0^thalassemia phenotype, is telomeric and removes MCS-R1, MCS-R2 and part of the MCS-R3 element. The other two, one associated with α^+^ and the other one with α^0^thalassemia phenotype, both showed a partial deletion of the MCS-R2 element. The molecular characterization of the breakpoints, the comparison of the new deletions’ phenotype with that of known α-thalassemia mutations, α-globin mRNA analysis from the heterozygotes, and functional analysis by luciferase assay, allowed to deepen our knowledge on the specific role of different parts of MCS-R2 and of the boundary regions in the long-range regulation of α-globin gene expression.

## Results

### Families and globin genotyping

Families A and D originate from Eastern Sicily, family B and C from Southern Italy. The complete hematological phenotype was obtained for several heterozygotes of the families A, C and D, and only for the proband of the family B (Table 1). Hb A2% and ferritin values were normal in all cases. The reduction of MCV (63.0–68.0 fL) and MCH (20.0–21.7 pg) indicated a severe defect of α-globin genes expression in the families A, B and C. Conversely, MCV (73.1–76.5 fL) and MCH (24.7–25.9 pg) values suggested a milder defect in 4 members of family D. Molecular genotyping revealed no point mutations or deletions of the α- and β-globin genes. Sequencing of the α- and β-globin genes showed that most heterozygotes for the deletions were heterozygous for the SNPs (Table 1); families A and B had the β-globin gene SNP HBB:c.9C>T (rs713040).

**Table 1:** Hematological data, SNPs and genotypes of the α1- and α2-globin genes of the members of the families with the new deletions. The SNPs associated with the deletions are marked in bold. nd: not determined; N: normal.

Family	**A**			**B**	**C**			**D**					
Sex/age (years)	M/53	F/52	F/26	F/75	M/56	F/53	M/16	M/59	F/53	M/32	M/28	M/40	F/08
Relationship	I.1	I.2	II.1	I.1	I.1	I.2	II.1	I.1	I.2	II.1	II.2	I.3	II.3
	father	mother	proband	proband	father	mother	proband	husband	proband	son	son	brother	niece
RBC (x10^12^/L)	6.35	4.75	6.19	5.77	5.19	5.72	6.55	4.74	5.07	5.38	5.19	5.64	4.35
Hb (g/dL)	13.0	14.5	13.0	12.5	15.9	12.0	13.7	14.2	12.5	13.9	13.4	14.0	12.4
Ht (%)	40.0	40.0	39.8	39.3	46.8	39.0	43.3	38.2	37.3	39.3	39.7	42.0	34.6
MCV (fL)	63.0	85.0	64.3	68.2	90.2	68.2	66.1	80.6	73.5	73.1	76.5	74.4	79.4
MCH (pg)	20.0	30.0	21.1	21.7	30.6	21.0	20.9	29.9	24.7	25.9	25.9	24.9	28.5
MCHC (L/L)	32.0	36.0	32.8	31.8	34	30.8	31.6	37.1	33.6	35.4	33.8	33.5	35.9
RDW (%)	nd	nd	16.3	17.1	13.2	16.4	17.2	14.2	14.9	13.6	14.4	14.8	12.8
HDW (g/dL)	nd	nd	3.53	nd	nd	nd	nd	2.91	2.72	2.84	2.53	2.68	2.52
Hb A2 (%)	2.3	2.3	2.3	2.4	nd	nd	2.2	2.5	2.5	2.8	2.8	2.4	2.8
Hb F (%)	1.0	0.5	1.1	0.3	nd	nd	0.2	0.2	0.2	0.4	0.1	0.4	0.6
Serum iron (mg/dL)	nd	nd	nd	94	59	47	42	nd	nd	nd	nd	nd	nd
Ferritin (ng/mL)	113	37	14	114	65	12	39	69	70	130	51	44	31
Bilirubin tot (mg/dL)	nd	nd	nd	0.64	nd	nd	nd	nd	nd	nd	nd	nd	nd
Bilirubin ind (mg/dL)	nd	nd	nd	0.52	nd	nd	nd	nd	nd	nd	nd	nd	nd
Ret (%)	nd	nd	nd	nd	1.8	0.8	0.2	nd	nd	nd	nd	nd	nd
DNA													
SNP *HBA2*+14C/G rs772829778	C/**G**	C/G	**G**	**C**	**C**	**C**	**C**	C/G	**C**	**C**/G	**C**/G	**C**	nd
SNP *HBA2*+861G/A rs2685121	**G**	G	**G**	G/A	nd	nd	**G**	G	**G**	**G**	**G**	**G**/A	nd
*SNP HBA1*+14C/G rs374054030	C/**G**	C	C/**G**	**C**	nd	nd	C	C	**C**	**C**	**C**	**C**	nd
heterozygote for α-thal deletion	(αα)ES	N	(αα)ES	(αα)FG	N	(αα)FG	(αα)FG	N	(αα)CT	(αα)CT	(αα)CT	(αα)CT	N

### Deletion detection

**Fig 2:**
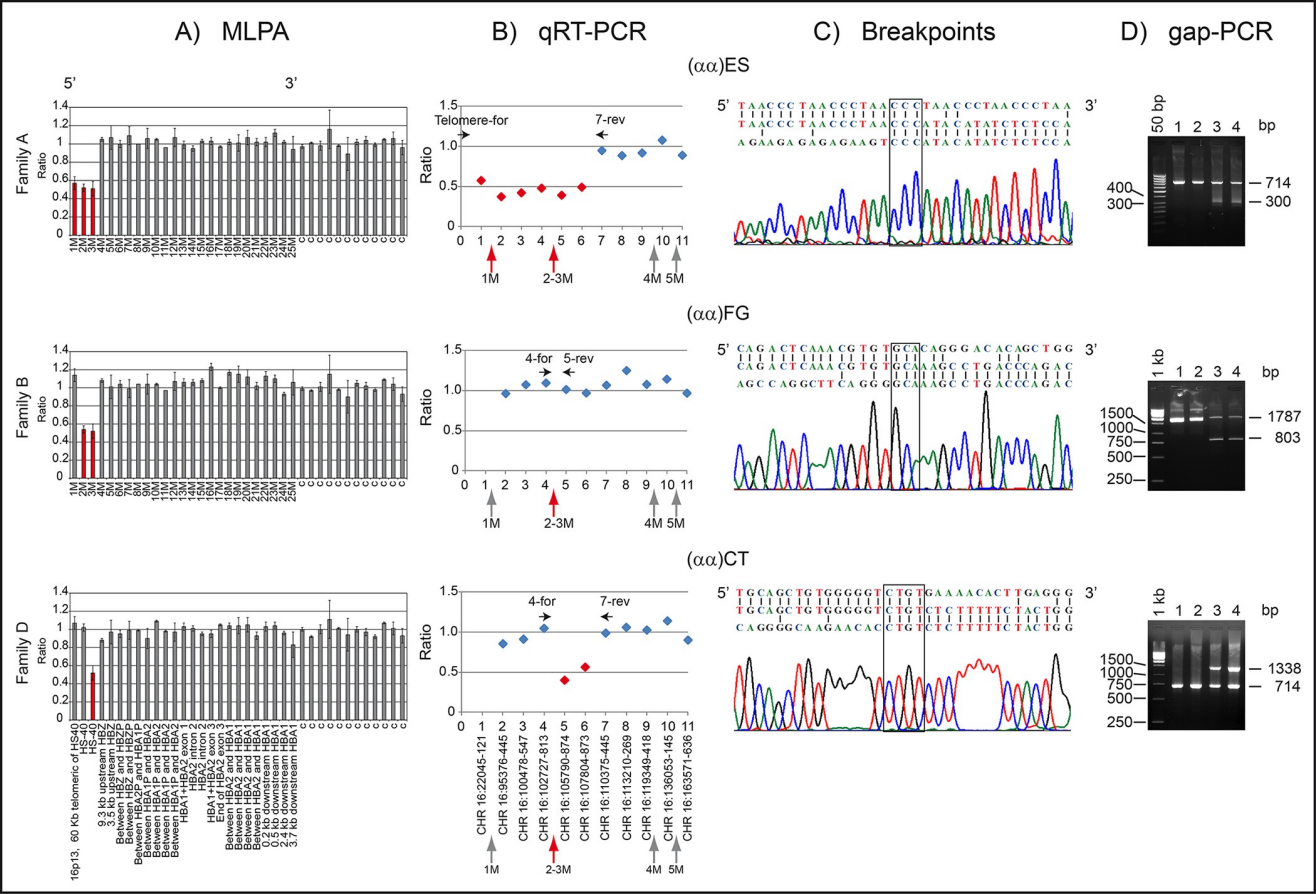
Molecular characterization of the three new α-thalassemia deletions. (A) Schematic representation of the MLPA ratio for 1M to 25M probes in heterozygotes for the new deletions. The relative position of the probes on chromosome 16 was reported below the scheme of the family D. Control (c) is the ratio of 12 probes from other chromosomes. (B) Schematic representation of the qRT-PCR ratio for 1 to 11 fragments, in heterozygotes for the new deletions. The primers (S1 Table) were specifically designed in a region of about 100 kb, as reported in Fig 1A. Fragments 10 and 11 (*HBA2*), in non-deleted regions, were used as control by testing 5 normal subjects. Horizontal arrows indicate the position and orientation of the primers utilized for long-range amplification. The position of the fragments relative to the MLPA probes (vertical arrows) is reported below each scheme. The number below the last scheme indicates the position of the fragment on the telomeric region of the chromosome 16. (C) Sequencing of the long-range gap-PCR fragments. Three sequences are reported in the upper part of each figure; in the middle the breakpoint sequence detected in the rearranged chromosomes, above and below respectively the regions at 5’ and 3’ of the breakpoint. The new deletions (αα)FG and (αα)CT most likely originated from non-homologous recombination events mediated by short sequences (highlighted with a box), common to both the extremities and probably causing the formation of loops. This is the case of “GCA” for the (αα)FG and “CTGT” for the (αα)CT. The deletion (αα)CT could also have been mediated by AT-rich element, 2 LINE/L1 and 4 SINE/Alu present in the deleted region. The new deletion (αα)ES had been most likely stabilized by the direct addition of telomeric repeats (TTAGGG)n to non-telomeric DNA, like the other telomeric deletions already described [24,25]. In favor of this hypothesis is the observation that the last three nucleotides (GGG), preceding the point of telomere addition, are complementary and in phase with the RNA template of human telomerase TTAGGG [24]. (D) Detection of the anomalous fragments amplified with the gap-PCR assays for the new deletions, set up for patient screening. On the left, the length (bp) of the ladder; 1, 2 normal subjects; 3, 4 heterozygotes of the new deletion as indicated above each picture. The 714 and 1787 bp bands are internal control; the 300, 803 and 1338 bp bands are specific for each deletion.

Probands from families A, B and D were analyzed by MLPA for the α- and β-globin clusters and for the upstream enhancer regions. MLPA analysis showed no rearrangement of the β-globin cluster. In contrast, three deletions were detected in the α-globin gene enhancer region (Fig 2A) [21–23].

In order to define the length of the deletions and the position of breakpoints sequences, eleven short regions were analyzed with qRT-PCR, (Figs 1A and 2B and S1 Table) [21–23].

### Identification of the breakpoints

Sequencing of the anomalous fragments obtained by long-range PCR, allowed the definition of the breakpoints (S2 Table and Figs 1 and 2).

**Family A**: Sequencing of the ~300 bp anomalous fragment reveals that the telomeric deletion (αα)ES (East Sicily), spans 120,180 bp, ending inside the MCS-R3 element at position +181 (NC_000016.10:g.1_(120178_120180)del120180). The (αα)ES chromosome was associated with the SNPs *ΗΒΑ2*+14G, *ΗΒΑ2*+861G, and the new polymorphism *ΗΒΑ1*+14G (Table 1). **Family B:** Long-range PCR showed two fragments, the normal of 1787 bp and an anomalous fragment of ~800 bp. The (αα)FG deletion, 984 bp-long, starts 679 bp upstream of the MCS-R2 element and ends inside this element, 51 bp upstream to its 3’ end (NC_000016.10:g.(112812_112814)_(113796_113798)del984). The (αα)FG chromosome was associated with the SNPs *ΗΒΑ2*+14C, and *ΗΒΑ2*+861G (Table 1).

**Family D:** Sequencing of the anomalous fragment of ~2500 bp indicated that the (αα)CT is 5058 bp-long and extends from position 113582_113585 to 118640_118643 on the chromosome 16 (NC_000016.10:g.(113582_113585)_(118640_118643)del5058). The (αα)CT chromosome was associated with the SNPs *ΗΒΑ2*+14C, and *ΗΒΑ2*+861G (Table 1).

The relative position and the extension of the new and the known deletions were reported in Fig 1A [13–20].

### Screening for the new deletions

During the writing of the manuscript, family C came to our attention showing α-thal phenotype, but the absence of the most frequent α- and β-thal mutations. The family native of Foggia, was analyzed with gap-PCR for the (αα)FG deletion (Fig 2D) revealing that proband and mother were positive, sequencing indicating that was the same deletion identified in proband B (Table 1).

### MCS-R1 genotyping

To assess the presence of mutations that could impact transcriptional rate outside the MCS-R2 region, sequencing of the MCS-R1 element was performed on a heterozygote for the (αα)CT (Family D, II.2), a heterozygote for the (αα)FG deletion (Family C, I.2), and a normal subject (Family C, I.1). All three subjects showed the same sequence characterized by homozygosity of two SNPs, rs798612 G and rs3056228 CTCTC (S2 Fig) both with high frequencies (about 87%) in the Caucasian population. These two SNPs have not been reported to be associated with a variation of expression.

### Genotype-phenotype relationship by ANOVA

**Fig 3:**
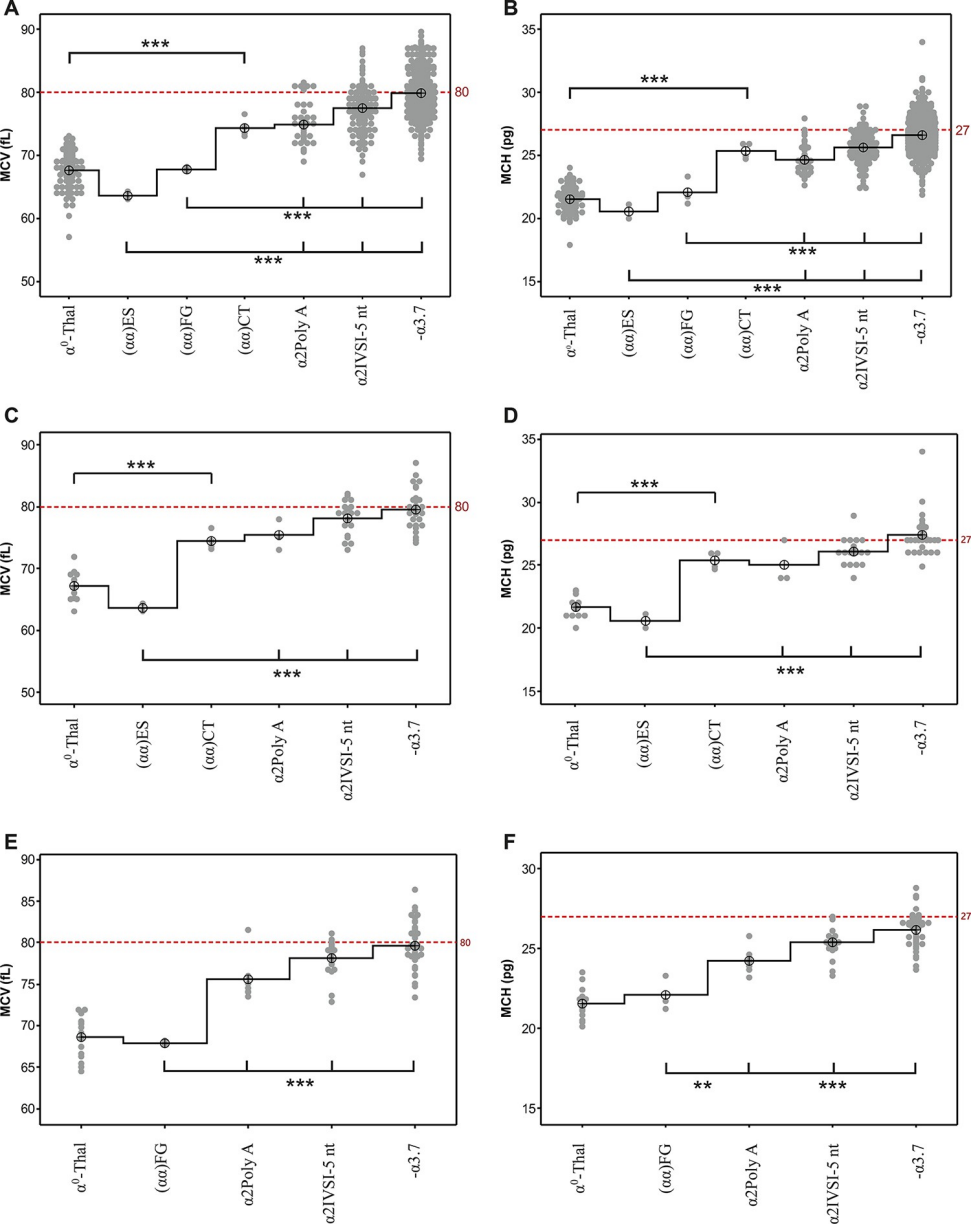
Individual value plot of haematological data from α-thalassemia heterozygotes. (A) and (B) 676 α-thalassemia heterozygotes with known mutations and normal iron metabolism were grouped in four classes in according with their genotype: α^0^thal (96 heterozygotes of--MED, -(α)^20.5^,--CAL deletions); -α^3.7^ (402 heterozygotes for the most frequent α+thal deletion); α2 IVS-I-5 nt (139 heterozygotes for α+thal point mutation); α2 polyA AATAAA>AATAAG (40 heterozygotes for α+thal point mutation). Heterozygotes for the new deletions were grouped into three classes based on genotype: (αα)ES (2 heterozygotes); (αα)FG (3 heterozygotes); (αα)CT (4 heterozygotes). (C) and (D) Heterozygotes for the new deletions (αα)ES (2 heterozygotes) and (αα)CT (4 heterozygotes) were compared with 64 heterozygotes of known mutations tested at the same hospital. (E) and (F) Heterozygotes for the new deletion (αα)FG (3 heterozygotes) were compared with 84 heterozygotes for known mutations, tested at the same hospital. On the X-axis the seven (A and B), six (C and D) and five (E and F) groups of α-thalassemia heterozygotes are reported; on the Y-axis the values of MCV (A, C, E) and MCH (B, D, F) are reported. The ⊕ symbol indicates the mean value of each group; the line is a mean connecting line; the mean value of each group was connected with a step connection function to highlight the differences between each group. The dashed line indicates the cutoff for the normal value. The *** denote that the Anova was statistically significant with test < 0.01.

Anova test revealed that the MCV and MCH of the (αα)CT heterozygotes showed comparable values to α^+^thal heterozygotes and statistically significant difference compared to α^0^thal heterozygotes. The hematological parameters of the (αα)ES and (αα)FG heterozygotes were comparable to those of the α^0^thal heterozygotes and showed statistically significant differences compared to α^+^thal heterozygotes (Fig 3A and 3B).

Comparison of hematological data of heterozygotes of the new and frequent mutations, from the same hospital, excluded any bias generated by the different hospitals’ device, as reported in Fig 3.

### mRNA analysis

**Fig 4:**
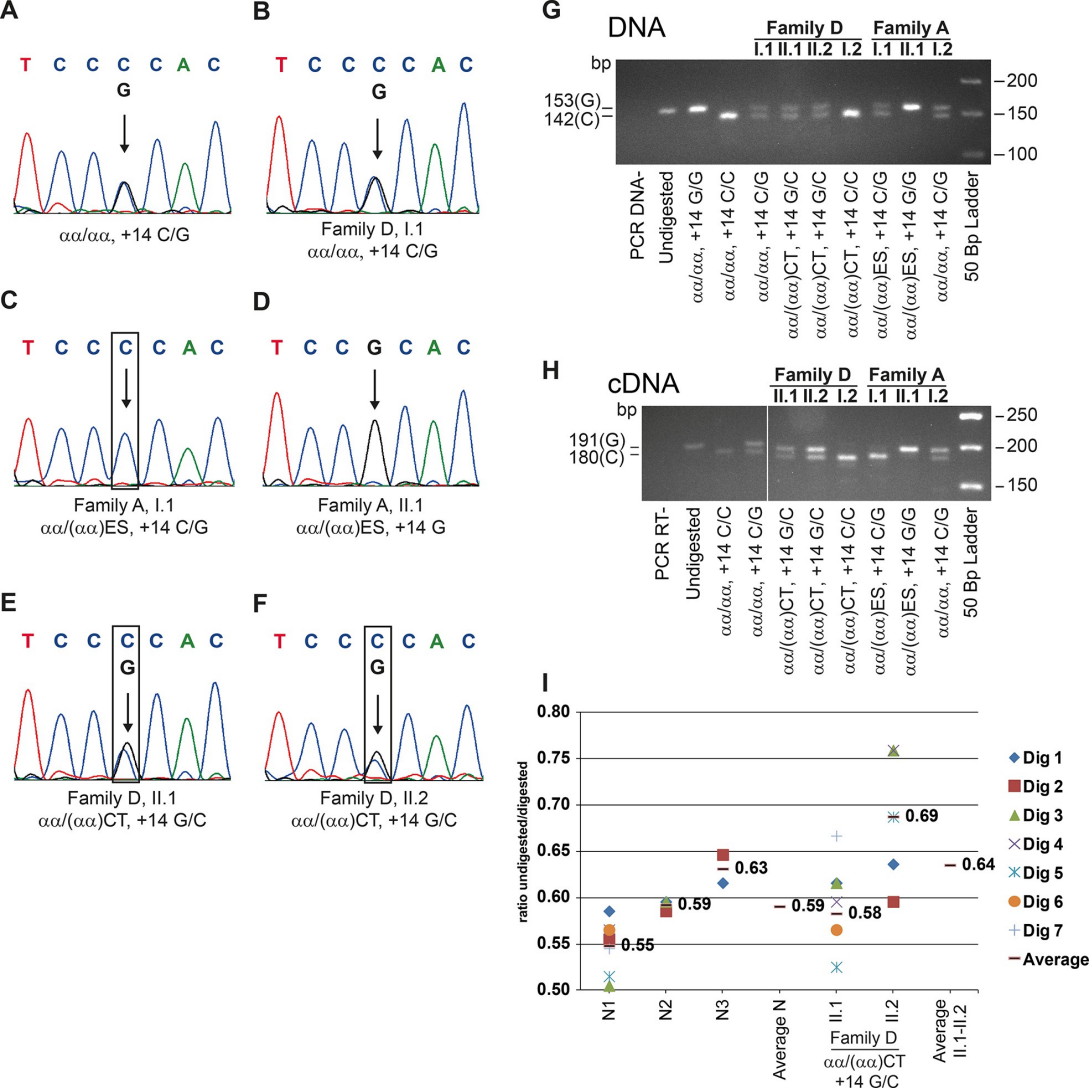
Sequencing and semi-quantitative analysis of the α2 globin DNA and cDNA from (αα)CT and (αα)ES heterozygotes. (A-F) cDNA sequences of the α2-globin genes from +11 to +17 of two normal subjects (A,B); two heterozygotes for the (αα)ES deletion (C,D), and two heterozygotes for the (αα)CT deletion (E,F); the arrow indicates the SNP +14. Below are reported: the family, the α-globin gene and SNP +14 genotype. (G) and (H) α2 globin gene DNA and cDNA analysis. The analysis was performed taking advantage of the fact that the *ΗΒΑ2*+14C/G can be distinguished for the presence/absence respectively of the NlaIV restriction site; the (αα)CT was associated with the *ΗΒΑ2*+14C; the (αα)ES was associated with the *ΗΒΑ2*+14G alleles. The DNA 153 bp long (G), and the cDNA 191 bp long (H) fragments were separated on a NuSieve agar gel, after digestion with NlaIV. Each family member is indicated as in Table 1. (I) Expression analysis by RE NlaIV recognizing the SNP +14C/G on the α2 globin cDNA. The analysis has been performed on three normal subjects (N1, N2 and N3) and on two heterozygotes for the (αα)CT deletion (II.1 and II.2 family D), the symbol - indicated the average value for each sample. The average of normal (N) and of mutant samples (αα/(αα)CT II.1-II.2) have been also reported. Dig = digestion.

Sequencing of the α2-globin cDNA was performed to detect the presence of the SNP *ΗΒΑ2*+14C>G in the families. Two normal subjects showed both C and G bases (Fig 4A and 4B); the father (I.1) of the family A, heterozygote for the (αα)ES deletion, showed only the presence of the base C (Fig 4C); two heterozygotes for the deletion (αα)CT showed both nucleotides C and G (Fig 4E and 4F).

The effects of the MCS-R2 deletions on the α-globin genes transcription were investigated by measuring the ratio of the mRNA synthesized by the two α2-globin genes through expression analysis by the restriction enzyme (RE) NlaIV recognizing the SNP +14C>G on the α2 globin cDNA [26–29].

The G/C ratio (undigested/digested) on the DNA of *HBA2*+14C>G heterozygotes, was 0.48±0.2 (Fig 4G, lanes 5–7,9,11). To obtain the best estimate of the two mRNA species in normal subjects, three normal *HBA2*+14C>G heterozygotes were selected and several RE analysis were performed, as reported in Fig 4I. The mean of the ratio G/C for each WT sample was respectively of 0.55, 0.59 and 0.63 with an average of 0.59 (0.41 base C) (Fig 4H, lanes 4,10; Fig 4I, N1-N3, Average N).

The only heterozygote for the (αα)ES deletion, heterozygote for the SNP *HBA2*+14C>G (Fig 4H, lane 8), did not reveal any trace of α2-globin cDNA associated with the G allele, but only cDNA from the normal cluster, associated with C; this means that the (αα)ES cluster does not produce α-globin mRNA at any detectable level. The daughter (homozygotes G) was non informative (Fig 4H, lane 9).

Analysis of the +14G and C species (undigested/digested ratio) in the two heterozygotes for the (αα)CT deletion, revealed a mean of 0.58 and 0.69, respectively, with an average of 0.64 (0.36 base C) (Fig 4H, lane 5–6; Fig 4I, II.1 and II.2, family D, Average II.1-II.2); considering that the deletion is associated *in cis* with the SNP +14C -which is the digested form- the analysis showed that the (αα)CT deletion displayed an expression level of about 56% (= digested/undigested*100) respect to the G allele. In the case of the heterozygotes for the SNP +14C>G, the expression level of the base C was approximately 70% with respect to base G (= digested/undigested*100). These data indicated that in the presence of the (αα)CT deletion the reduction of expression was mild, with a decrease of about 15% on both the α-globin genes.

### Expression analysis of MCS-R2 constructs

An ongoing challenge in the study of **α**-globin genes is to characterize the activity of their regulatory elements. The identification of the two novel small deletions involving the MCS-R2 element and showing unequal phenotype, gave us the opportunity to study the enhancer activities of MCS-R2 regions using luciferase reporter assays in the erythroid K562 cell line.

### (αα)FG deletion constructs

The luciferase data from K562 cells experiments, normalized to MCS-R2wt indicated that MCS-R2sh showed comparable activities respect to MCS-R2. Instead, the two constructs Del(αα)FG-495G/A showed the higher enhancer activity that was about double that of MCS-R2. The differences were statistically significant.

The comparable enhancer activities of the constructs Del(αα)FG-495G and A (1.91 and 2.09) indicated that this SNP is not involved in the interaction with transcriptional factors.

**Fig 5:**
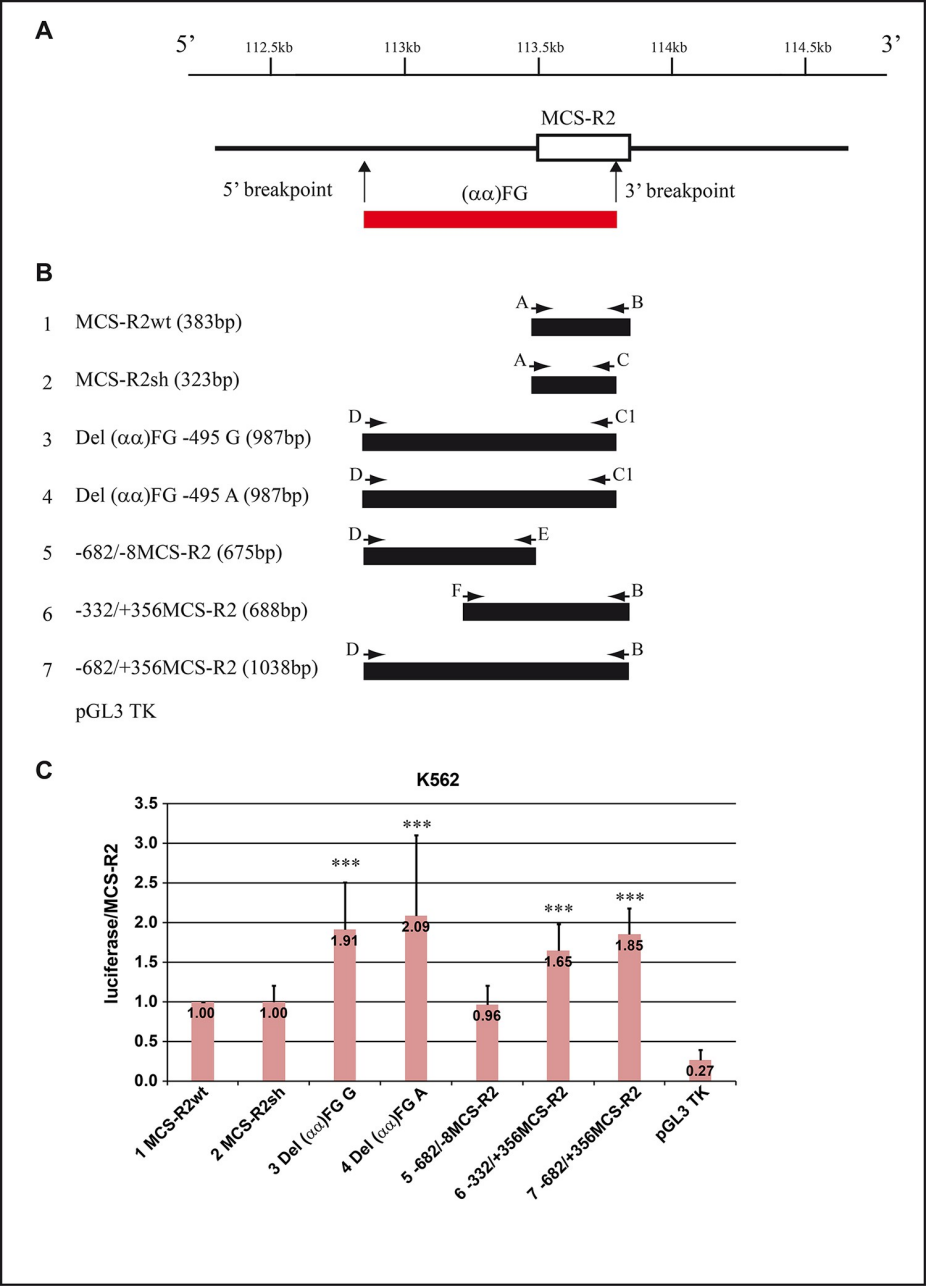
Luciferase assay on enhancer vectors from the (αα)FG deletion. (A) In the upper part the normal chromosome with the position of MCS-R2 element. In the bottom part the position and extension of the (αα)FG deletion, the vertical arrows indicating the position of the breakpoints. (B) Representation of the seven constructs cloned for the functional analysis of the region contained in or close to the (αα)FG deletion. On the left the number, the name and the length, in the middle position and extension of each cloned fragment. The horizontal arrows represent the position and orientation of the primers used for the amplification. (C) Histogram of the luciferase activity average, with deviation standard, normalized to the MCS-R2wt activity tested in K562 cell lines. The *** indicate that the T student was statistically significant with T test < 0.01.

The analysis of the subsequent three constructs in K562 cells indicated that the 682 bp upstream region had enhancer activity (0.96) comparable to MCS-R2wt, and that the presence of the boundary region *in cis* to MCS-R2 increase the enhancer activity in relation to the boundary region length starting from 1.65 up to 1.85 (Fig 5C).

As expected, the luciferase activity of the pGL3 basic Tk vector, tested as negative control, was 0.27 of the MCS-R2 activities, value congruent with a basic promoter activity.

### (αα)CT deletion constructs

**Fig 6:**
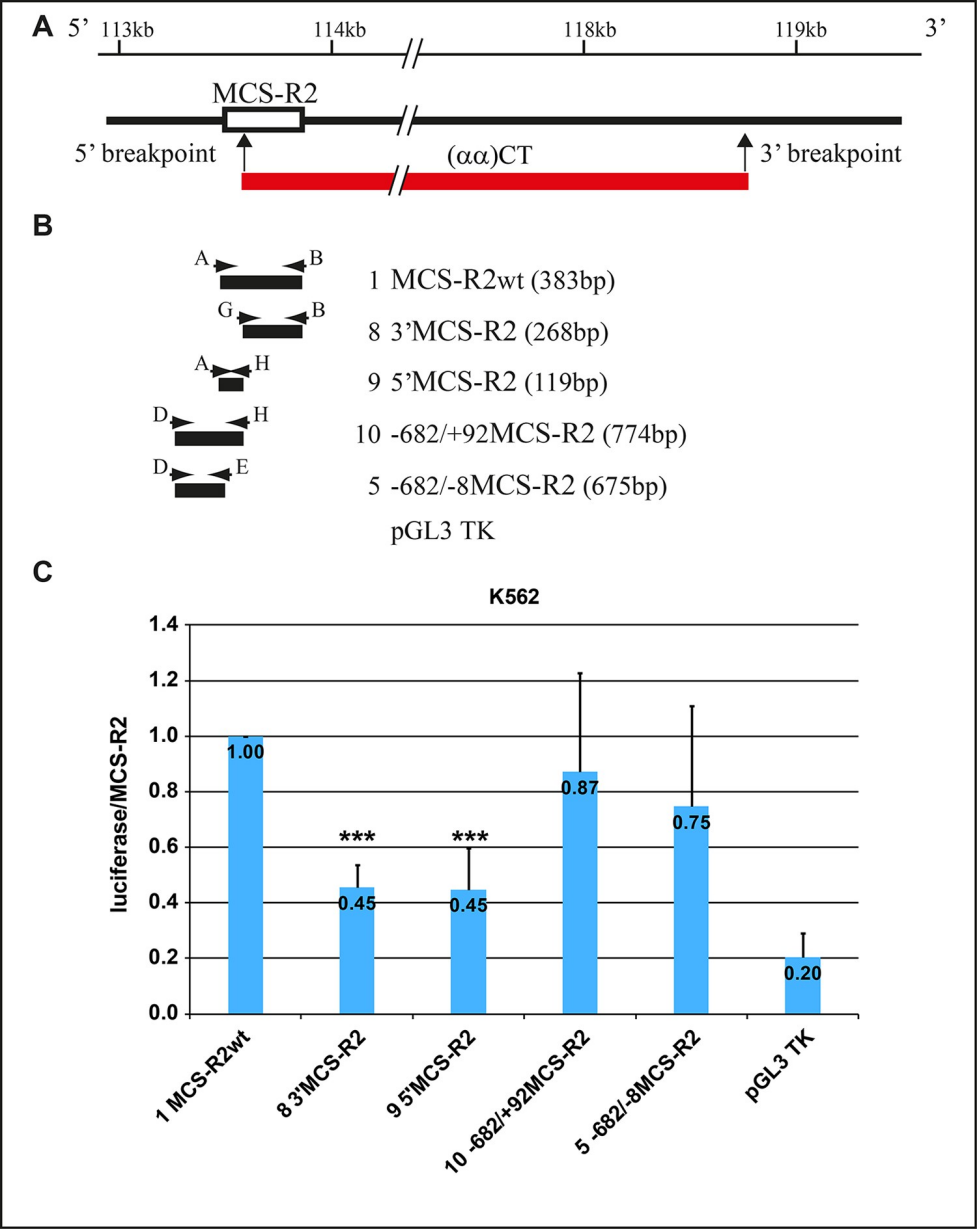
Luciferase assay on enhancer vectors from the (αα)CT deletion. (A) In the upper part the normal chromosome with the position of MCS-R2 element. In the bottom part the position and extension of the (αα)CT deletion, the vertical arrows indicating the position of the breakpoints. (B) Representation of the five constructs cloned for the functional analysis of the region contained in or close to the (αα)CT deletion. On the left, the number, the name and the length, in the middle the position and extension of each cloned fragment. The horizontal arrows represent the position and orientation of the primers used for the amplification. (C) Histogram of the luciferase activity average, with standard deviation, normalized to the MCS-R2wt activity tested in K562 cell lines. The *** indicate that the T student was statistically significant with T test < 0.01.

The deletion (αα)CT, 5058 bp-long, starts at position +93 of MCS-R2 (Figs 1C and 6A). The association with a mild α-thalassemia phenotype suggested that the first part (93 bp) of MCS-R2 has a partial positive effect on the long-range regulation of the α-globin genes.

The analysis of the luciferase assay, normalized to the MCS-R2wt construct, revealed that in K562 cells the 3’ and 5’MCS-R2 fragments showed a comparable enhancer activity (0.45 and 0.45), but lower than the MCS-R2wt element.

The construct n.10 showed enhancer activity (0.87) further increased respect to the short 5’MCS-R2 (n.9) (0.45) and to the construct n.5 (0.75).

The Fig 6C shows that the presence of the boundary region supports the 5’MCS-R2, showing an enhancer activity of 0.87 (construct n.10), comparable to MCS-R2wt.

As expected, the pGL3 TK basic vector, transfected as negative control, showed a luciferase activity of 0.20 respect to the MCS-R2 element.

In conclusion, in K562 cells, the MCS-R2 element is necessary to obtain a higher expression of the luciferase in presence of its 682 bp upstream region. This is in agreement with α-thal phenotype of our shorter two deletions. In patients lacking of the 987 bp, heterozygotes for the (αα)FG, the expression of the α-globin genes is severely affected, while in the heterozygotes for the (αα)CT deletion the presence of only 93 bp of MCS-R2 is enough to drive a moderate gene expression in presence of its 5’ upstream region.

## Discussion

The identification of natural mutants in the human population is the elective approach to determine the role of individual regulatory elements *in vivo*, by providing their expression in the physiological human erythroid environment.

In this paper we report the characterization of three new deletions, positioned in the MCS-R2 region, named (αα)ES, (αα)FG and (αα)CT (Figs 1 and 2).

Both the MCV and MCH parameters of the (αα)ES and (αα)FG, and those of the (αα)CT heterozygotes resulted of α^0^-thalassemia and α+thalassemia phenotype, respectively, when compared to a control cohort (Table 1 and Fig 3).

Transcription analysis on reticulocyte cDNA indicated that the telomeric deletion (αα)ES completely inactivated the *in cis* expression of the α-globin genes (Fig 4C and 4H). Conversely, the (αα)CT heterozygotes showed a high level of expression of the C isoform, *in cis* to the deletion (Fig 4E, 4F and 4H).

To explain the high level of expression of the G isoform in normal +14C/G heterozygotes, one hypothesis could be that the C isoform is less expressed than the G isoform probably due to the different interaction with transcription factors. To corroborate this hypothesis, it might be useful to run the analysis on a larger number of heterozygotes *ΗΒΑ2*+14C/G α-globin mRNA samples, but incidentally, we did not have any other samples. It may also be informative to perform a transcriptional analysis in cell system using expression vectors. These additional approaches could be explored in the future, but in our opinion the relevant finding is that the expression rate of the C isoform of about 56% (= 36/64%) respect to the G isoform in the heterozygote for (αα)CT is significantly high in presence of only the first 93 bp of the MCS-R2 enhancer element. In these first 93 bp (Fig 1C), the “CTATCA” sequence, binding site for the erythroid specific nuclear factor GATA-1(a), and the “GGTGG” sequence, putative binding site for the GT ubiquitous transcription factors, are present at position +16 and +64, respectively [6–8]. Hence, our data confirm the importance of this short region in modulating the expression of α-globin genes. The dissection of the sequential order in which transcription factors are recruited to the locus led to the conclusion that MCS-R2 has multiple roles as the recruiter of PolII and of key transcription factors at the promoter, and starter of the loop structure involving several remote regulatory elements [9,30].

Our analysis of the (αα)CT deletion highlights that this deletion preserves these functions.

The three (αα)FG heterozygotes suffered of severe microcythemia (Table 1) associated with a severe α-globin genes inactivation (Fig 5). Unfortunately, the (αα)FG was not associated with specific SNP useful for performing a transcription analysis.

To understand more about the role of the MCS-R2 boundary regions, the study of these sequences in expression vectors has been very convenient. The constructs analysis highlighted that the 680 bp upstream to MCS-R2 were able to duplicate the enhancer activities in K562 erythroid cells, preserving a good enhancer activity even in the absence of MCS-R2 (Fig 5). Analysis of the (αα)CT deletion revealed that the first 93 bp, still present in the deleted chromosome, are able to drive a consistent enhancer activity of the α-globin gene cluster supported by the 5’MCS-R2 region (Fig 6).

The MCS-R1 element has been supposed to contribute 10% of α-globin gene expression. Its sequence analysis ruled out the presence of mutations that could impact on the transcriptional rate of the (αα)CT and (αα)FG deletions (S2 Fig).

To acquire useful data on the function of specific sequences, we compared the phenotype of the three new deletions with that of three short deletions newly described, removing almost only the MCS-R2 element and part of the upstream boundary region: (αα)#18 (6710 bp starting from -3500); (αα)ALT (3361 bp starting from -300); the (αα)JX (742 bp starting from -333) [10,16–20]. The extent of the deletions and the heterozygote’s phenotype, (Fig 1 and Table 2) support the hypothesis of an enhancer activity present in the boundary region upstream of MCS-R2.

**Table 2:** Haematological data of the heterozygotes for the (αα)ALT, (αα)JX, and (αα)#18 deletions.

Family n.	Gender/age	RBC (10^12^/L)	Hb (g/dL)	MCV (fL)	MCH (pg)	HBA_2_ (%)	HbH (%)	HbF (%)	Ret (%)	Ferritin (ng/mL)	α/β ratio	α-globin genotype	Ref
1	M	6.5	14.7	70.2	22.7	2.2	nr	nr	nr	nr	nr	αα/(αα)ALT	[17]
2	M/46	nr	14.0	73.0	24.0	2.7	nr	nr	nr	139	nr	αα/(αα)ALT	[16]
3	M/father	5.0	10.3	64.7	20.5	1.3	present, nq	0.5	nr	nr	nr	(αα)ALT/(αα)ALT	[17]
	F/dauther	5.7	13.6	74.2	23.9	2.3	nr	nr	nr	nr	nr	αα/(αα)ALT	
4	F/mother	nr	11.1	72.7	23.8	nr	nr	nr	nr	nr	nr	αα/(αα)ALT	[18]
	M/11 son*	nr	(6.0-8.0)	69.3	20.2	nr	30	nr	4.4	nr	0.61	--tel del/(αα)ALT	
5	M/father	nr	15.7	73	24	nr	nr	nr	nr	nr	nr	αα/(αα)ALT^&^	[10]
	F/26 dauther	5.8	6.9	40	12.7	nr	present^§^	nr	2	45	0.3^#^	--SEA/(αα)ALT^&^	
6	M/father	nr	13.0	74.8	24.8	nr	nr	nr	nr	nr	nr	αα/(αα)JX	[20]
	F/adult dauther	nr	10.9	76.0	24.7	nr	nr	nr	nr	nr	nr	αα/(αα)JX	
	F/adult dauther	nr	11.8	74.3	24.6	nr	nr	nr	nr	nr	nr	αα/(αα)JX	
	M/adult son	nr	14.9	76.0	24.8	nr	nr	nr	nr	nr	nr	αα/(αα)JX	
7	M/granfather	nr	14.2	75.8	25.0	nr	nr	nr	nr	nr	nr	αα/(αα)JX	[19]
	F/mother	nr	10.8	75.6	24.2	nr	nr	nr	nr	nr	nr	αα/(αα)JX	
	F/two mounths**	nr	6.9	54.0	23.6	nr	nr	nr	nr	nr	nr	--SEA/(αα)JX	
8	M/24	nr	13.3	69.0	23.0	2.9	nr	7.4	nr	114	nr	αα/(αα)#18	[16]

nr = not reported; nq = not quantized; * Hb level was between 6.0 and 8.0 gr/dL and the patient needed periodical red blood cell transfusions; ^&^β-globin genotype: β^A^/β^E^; ^§^ HbH inclusions were present in 30.7% of red blood cells; ^#^ A/B ratio from peripheral blood was double than from erythroid precursors in culture; ** the patient received the first transfusion at the age of two months, then she became transfusion-dependent at an interval of every 1 month.

The hematologic profile of (αα)ALT and (αα)JX heterozygotes indicates that the mean MCV value was of 72.62±1.47 and 75.42±0.7, respectively, comparable to that of the (αα)CT (MCV 74.37±1.52) and higher than (αα)FG (MCV 67.5±1.21) heterozygotes. (αα)JX and the (αα)ALT have been described in two and in five families, respectively, identified in different hospitals, and in all the cases a mild phenotype was observed in heterozygotes [16–20]. Another important piece of information is that compound heterozygous or homozygote for (αα)JX and (αα)ALT (Table 2) show an intermediate thalassemia phenotype, instead of hydrops fetalis, indicating that the α-globin genes, *in cis* to the (αα)JX and (αα)ALT deletions, are not completely silenced [16–20]. The difference between these two deletions and the novel deletion (αα)FG is a 350 bp long region spanning from -682 to -333 bp, 5’ to MCS-R2. This region is retained in the (αα)JX and (αα)ALT while it is missing in (αα)FG. The hematological and luciferase data allowed us to hypothesize that the upstream region of about 350 bp is most likely able of binding transcription factors to direct the synthesis of α-globin chains *in cis* to the (αα)JX and the (αα)ALT deletion.

All these data allow us to conclude that both (αα)JX and (αα)ALT deletion are able to support the synthesis of α-globin genes in a higher way than (αα)FG; in other words, we can say that (αα)FG delimits further the region showing an enhancer activity, in accordance with a more severe phenotype. As shown in Fig 1B the (αα)FG spans 350 bp, compared to the shorter deletion (αα)JX, the upstream sequence required for full α-globin genes expression [10,16–20].

Instead, the only heterozygote for the (αα)#18, characterized by deletions of a consistent boundary MCS-R2 regions containing all the examined upstream region of 680 bp, showed a more severe phenotype [16].

New acquisitions on regulatory regions of the α-globin cluster are very useful considering that the editing of the α-globin enhancer has been proposed for the treatment of β-thalassemia [31].

The identification of an increasing number of new or rare deletions in the MCS-R2 enhancer element suggests to extend the search for deletions to it in case of unexplained Hb H phenotype [32]. Given the important role that α-globin genes play as modifiers genes on the phenotype of β-globin variants, this further analysis may reveal new data helping in the clinical classification [33,34].

In conclusion, the new deletions provide important insights into the role of the MCS-R2 element and of the surrounding region. We observed total silencing of α-globin genes associated with the telomeric (αα)ES deletion and a significantly high expression in case of (αα)CT, the first known deletion that removes part of MCS-R2. Moreover, the (αα)FG is the smallest deletion with a severe phenotype that functionally delimit the enhancer region. Combined deletion analysis showed that the 5’ boundary region of MCS-R2, absent in (αα)FG and present in the (αα)CT, plays a functional role of enhancer in MCS-R2 deletions. The comparison with other described deletions confirms these data. Interestingly, the (αα)JX, by removing MCS-R2 entirely, has a less severe phenotype than (αα)FG, which removes not only MCS-R2 almost entirely, but also a 679 bp region upstream of it. Based on our new data, we conclude that an enhancer element useful to increase expression of the α-globin genes is present in this 679 bp region. In other words, duplicate sequences that are not active under normal conditions, could ensure the function of regulatory elements even in case of deletions of one of them in order to preserve key functions. [9,20]

## Methods

### Ethics statement

Ethical approval of the study protocol was obtained from the Ethics Committee “Università Federico II–Azienda Ospedaliera di Rilievo Nazionale (A.O.R.N.) Antonio Cardarelli” of Naples (307/2016, 225/2019). Patient consent was waived under the approval of the Ethics Committee (307/2016) using data de-identification.

### Patients and hematological data

The probands and their families were selected by the Thalassemia Centers collaborating in this study among those referred to them for the random screening and hematological diagnosis (Table 1). Blood counts and Hb analysis were obtained using standard methods.

### DNA molecular analysis

The screening for the most common α-thalassemia deletions (-α^3.7^ -α^4.2^, (α)α^5.3^,--Med, -(α)^20.5^,--CAL) was carried out with the gap-PCR method. The α-thalassemia point-mutations were screened with multiplex-ARMS, DGGE or DNA sequencing [35,36]. The α2 or α1-globin genes were sequenced from positions -181 to +884 (α2) and +894 (α1) [37].

The β-thalassemia point-mutations were analyzed with the ARMS method or gap-PCR or with DNA sequencing, carried out in all cases with Automated Cycle Sequencing (3100 Genetic analyzer, Applied Biosystems) [37].

### MLPA, qRT-PCR and long range-PCR

The molecular characterization of the new deletions of the α-globin gene cluster was performed by Multiplex Ligation Probe Amplification (MLPA) assay, qRT-PCR, long-range PCR and sequencing analysis for the definition of the breakpoints, as previously reported (S1 and S2 Tables) [21–23,38–40].

Two MLPA kits (MRC-Holland, Amsterdam, the Netherlands) were used for the identification of deletion or triplication in the α-globin gene cluster (SALSA MLPA KIT P140 HBA) and for the identification of deletions in the β-globin gene cluster (SALSA MLPA KIT P102 HBB). MLPA products were separated by ABI-3130XL Genetic Analyzer (Applied Biosystems, Foster City, CA, USA), quantified with the Coffalyser software (MRC-Holland), and compared with a DNA pool from normal subjects, as previously reported [21–23,38,39]. qRT-PCR was performed with the ABI 7900HT System and the Power SYBR-Green PCR-Master mix (Applied Biosystems) with primers chosen outside repeated sequences (see S1 Table). The beta-2-microglobulin was used as reference gene [40].

The pair of primers for the long range-PCR was selected among those used for the qRT-PCR and was from the normal regions close to 5’ and 3’ of each breakpoint (S2 Table, Fig 2B) [21–23].

### MCS-R1 analysis

The sequence of the MCS-R1 (HS-48) element was carried out using primers and conditions reported in S2 Table.

### mRNA analysis

Qualitative and semi-quantitative analysis of the α2-globin cDNA was performed as already reported [26–29]. To analyze the SNP α2+14C>G (rs772829778) at mRNA level, it was designed a short 13 bp oligonucleotide 5’-ACTCTTCTGGTCC-3’ (α-globins mRNA position +1/+13), the first 7 nucleotide (underlined) being of Locked Nucleic Acids (LNA) type (ROCHE DIAGNOSTICS SPA, Milan-Italy) to gain a melting temperature of 58°C. Digestion of DNA and cDNA amplimers (S2 Table) with the RE NlaIV (Fermentas Life Science, Massachusetts, USA), able to recognize the α2+14C, highlighted the rate of expression of the two α2-globin alleles in heterozygous subjects. The digested fragments were separated on a NuSieve 3:1 agarose 3.5% gel (FMC, Rockland, Maine, USA), and a semi-quantitative analysis of the bands was performed with the Kodak software Carestream MI, as already reported [26–29]. A corrective factor was used to normalize for the loss of a shorter band of 11 bp and the efficiency of digestion of the NlaIV was performed (S1 Fig). The data on cDNAs samples were confirmed two times on different mRNA extractions.

### Firefly-renilla luciferase reporter gene assay

The enhancer activity of different parts of the MCS-R2 element was studied by using pGL3-TK Basic Vector (Promega, Madison-WI) cloning constructs upstream to the Luciferase reporter gene, and by transient co-transfection with pGL3 Renilla vectors, in K562 cell lines (S3 Table) [27,41,42]. The pGL3-TK Basic Vector was transfected as negative control.

The deletion (αα)FG, 984 bp-long, started 679 bp upstream MCS-R2 and ended inside this element, 51 bp upstream to its 3’ end (Figs 1 and 5A). To analyze the (αα)FG deletion in a first instance, four constructs were generated (Fig 5B and S3 Table): MCS-R2wt (383 bp); MCS-R2sh, the deleted portion lacking the last 50 bp (323 bp); Del(αα)FG-495G/A, two deleted constructs (987 bp) polymorphic in position -495G>A. Then, we focused the attention on the potential enhancer role of the 682 bp region upstream to MCS-R2, which was analyzed in three other constructs: alone (-682/-8); associated to MCS-R2 in full length (-682/+356); and shortened (-332/+356) (Fig 5B).

To analyze the (αα)CT deletion we started cloning three genomic constructs upstream to the luciferase gene (S3 Table): MCS-R2wt (-27/+356, 383 bp); the 3’MCS-R2 deleted portion (+89/+356, 268 bp); the 5’MCS-R2 portion still present in (αα)CT deletion (-27/+92, 119 bp) (Fig 6). In order to elucidate the sequences with enhancer activities, we subsequently analyzed the region upstream to 5’MCS-R2. Therefore, the fragment n.10 was cloned, starting 682 bp upstream to MCS-R2 at the beginning of Del(αα)CT (-682MCS-R2/+93MCS-R2, 774 bp). The new construct was compared with construct n.5, (-682/-8MCS-R2), already analyzed for the (αα)FG.

Detailed materials and methods are described in the S1 Methods.

### Genotype-phenotype relationship

A control cohort of 676 α-thalassemia heterozygotes (>18 years), previously molecularly characterized, were extracted from a Proprietary Database at IGB, for the genotype-phenotype correlation. MCV and MCH of the patients were grouped in four classes, in relation to the genotype (α^0^ and α^+^thal heterozygotes) and compared with those of the new deletions’ heterozygotes. The genotypes of the four classes are reported in the legend of Fig 3.

To overcome any bias generated by the different hospitals’ devices, the haematological data of the (αα)ES, of the (αα)CT heterozygotes (Fig 3C and 3D) and of the (αα)FG heterozygotes (Fig 3E and 3F) were respectively compared with heterozygotes for known mutations tested at the same hospital.

### Statistical analysis

Statistical analysis was conducted with the software Minitab v.16 using the One-way ANOVA method to compare the hematological data from the heterozygotes for the three new deletions, with that of previously studied α^+^ or α^0^thalassemia heterozygotes. P<0.05 was considered statistically different [22].

Results of luciferase assay are reported as mean ± standard deviation. The Student’s t-test was used for the estimation of statistical significance. Significance for statistical analysis was defined as a p < 0.01.

### Database

All the hematological data, the experimental results and the family relationships were collected in a Proprietary Database at IGB, developed on Microsoft Visual Fox platform, and interfaced with external software such as Microsoft Excel and Microsoft Word [43].

## Supporting information

S1 Fig NlaIV digestion efficiency. cDNA amplicomer fragment of 191 bp, from an α2+14 C/C subject, digested by 10 U of NlaIV restriction enzyme, and separated on a 3.5% NuSieve agarose gel. The α2+14 C/C has the NlaIV restriction site GGT’CCC, generating a shorter cDNA band of 11 bp -not visible because the fast migration- and the 180 bp band. The recommended protocol for digestion of PCR products indicated to use 10 U of enzyme with about 100–500 ng of DNA. To test the NlaIV digestion efficiency increasing amount of PCR products, from 50 to 300 ng, have been used. All the samples showed 100% digestion. Lane 1: 50 ng amplicomer; Lane 2: 100 ng amplicomer; Lane 3: 200 ng amplicomer; Lane 4: 300 ng amplicomer; Lane 5: undigested amplicomer sample; Lane 6: Low Range Ladder. The fragments’ lengths are reported on the right.
(TIF)

S2 Fig MCS-R1 DNA sequence analysis. (A) (B) (C) Forward sequence of the MCS-R1 element, showing the rs798612 (A/G/T). (A) Heterozygote for the (αα)CT deletion (Family D, II.2); (B) Normal subject (Family C, I.1); (C) Heterozygote for the (αα)FG deletion (Family C, I.2). (D) (E) (F) Reverse sequence of MCS-R1 showing the rs3056228 dupTC CTC/CTCTC. (D) Heterozygote for the (αα)CT deletion (Family D, II.2); (E) Normal subject (Family C, I.1); (F) Heterozygote for the (αα)FG deletion (Family C, I.2). The sequences of the three samples were identical to the MCS-R1 reference NC_000016.10 (104622–105621) with the exception of the two SNPs reported above, for which the samples were homozygotes G (0.873665 in European) and CTCTC (0.86954 in European) respectively.
(TIF)

S1 Table Primers for the qRT-PCR assays. The position on the gene sequence was defined according to the Gene Bank sequence accession number NT_037887.4.
(DOCX)

S2 Table Primers and experimental conditions for the long-range, gap-PCR and sequencing for the definition of the breakpoints at the new deletions and for the semiquantitative analysis of the α2 globin gene cDNA and DNA. The position of the primers on the gene sequence was defined according to the Gene Bank sequence accession number NC_000016.10.
(DOCX)

S3 Table Oligos designed for the directional cloning. The sequence and the position of the oligos, respect to the MCS-R2 element or to the deletions reported in the present article, and the length of the clone are reported. Upstream of the 5’ primer was added the sequence *ggtacc*, recognized by the KpnI enzyme, and downstream of the 3’ primer was added *gctagc*, recognized by the NheI enzyme.
(DOCX)

S1 Methods Supplementary Methods. (PDF)

S1 Data Excel spreadsheet with numerical raw data underlying Figs 3A, 3B, 3C, 3D, 3E, 3F, 4I, 5C and 6C. (XLSX)
