# Supplementary figures and images for: Functional analysis of three new alpha-thalassemia deletions involving MCS-R2 reveals the presence of an additional enhancer element in the 5’ boundary region
Source: PLoS Genet. 2023 May 22;19(5):e1010727. doi: 10.1371/journal.pgen.1010727 (PMC10202303; doi:10.1371/journal.pgen.1010727)

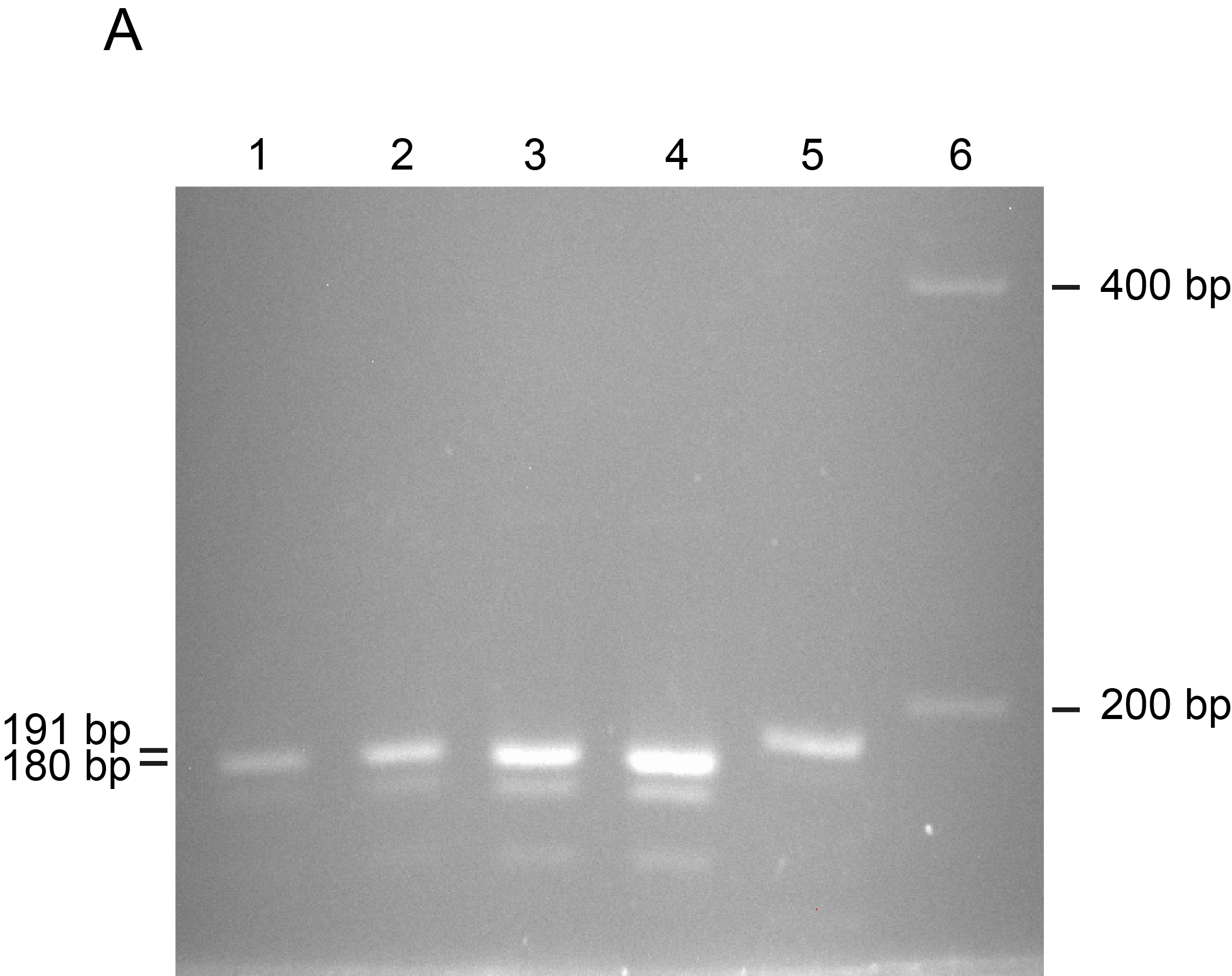

Supplement: S1 Fig — cDNA amplicomer fragment of 191 bp, from an α2+14 C/C subject, digested by 10 U of NlaIV restriction enzyme, and separated on a 3.5% NuSieve agarose gel. The α2+14 C/C has the NlaIV restriction site GGT’CCC, generating a shorter cDNA band of 11 bp -not visible because the fast migration- and the 180 bp band. The recommended protocol for digestion of PCR products indicated to use 10 U of enzyme with about 100–500 ng of DNA. To test the NlaIV digestion efficiency increasing amount of PCR products, from 50 to 300 ng, have been used. All the samples showed 100% digestion. Lane 1: 50 ng amplicomer; Lane 2: 100 ng amplicomer; Lane 3: 200 ng amplicomer; Lane 4: 300 ng amplicomer; Lane 5: undigested amplicomer sample; Lane 6: Low Range Ladder. The fragments’ lengths are reported on the right. (TIF) [file pgen.1010727.s001.tif]

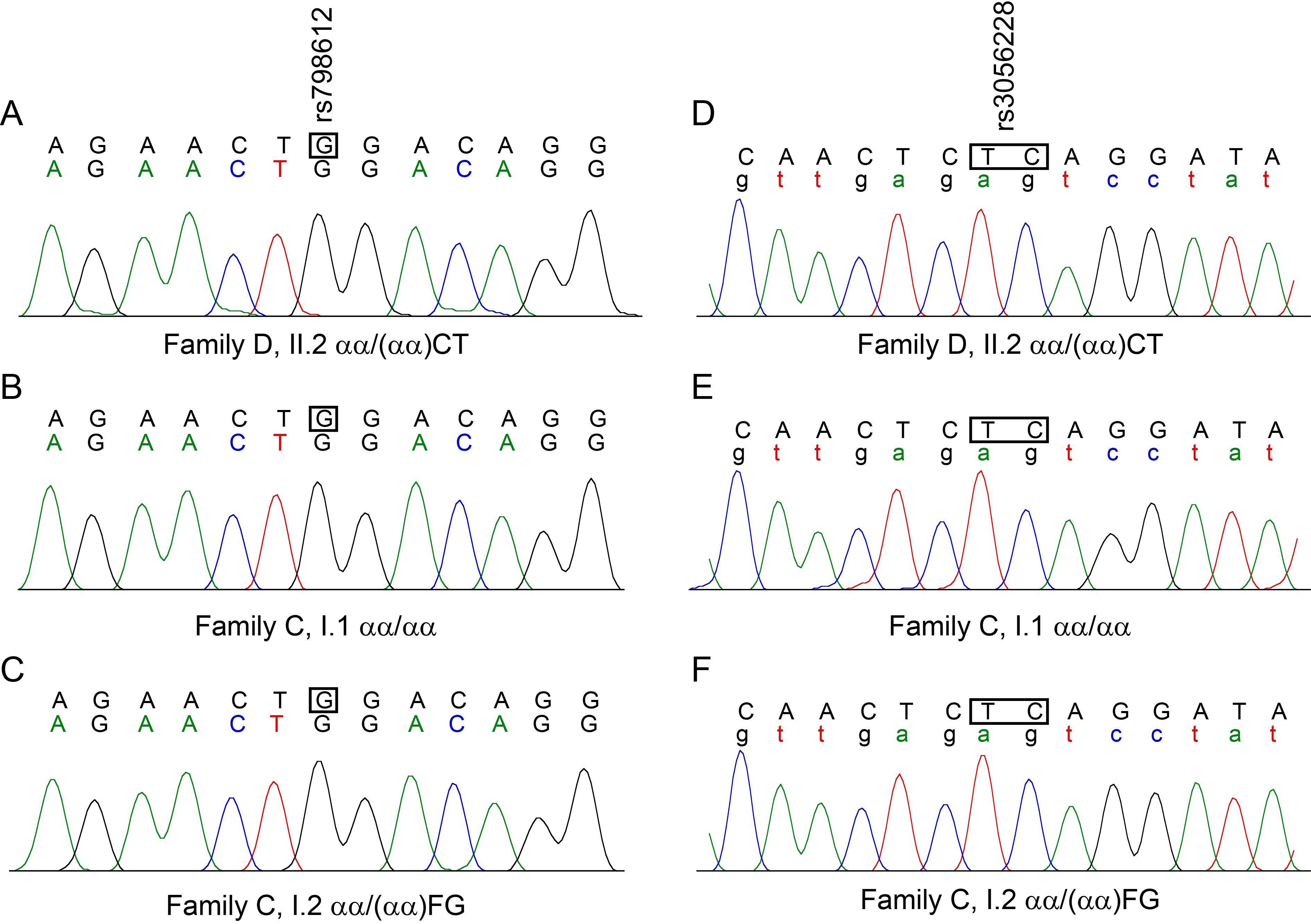

Supplement: S2 Fig — (A) (B) (C) Forward sequence of the MCS-R1 element, showing the rs798612 (A/G/T). (A) Heterozygote for the (αα)CT deletion (Family D, II.2); (B) Normal subject (Family C, I.1); (C) Heterozygote for the (αα)FG deletion (Family C, I.2). (D) (E) (F) Reverse sequence of MCS-R1 showing the rs3056228 dupTC CTC/CTCTC. (D) Heterozygote for the (αα)CT deletion (Family D, II.2); (E) Normal subject (Family C, I.1); (F) Heterozygote for the (αα)FG deletion (Family C, I.2). The sequences of the three samples were identical to the MCS-R1 reference NC_000016.10 (104622–105621) with the exception of the two SNPs reported above, for which the samples were homozygotes G (0.873665 in European) and CTCTC (0.86954 in European) respectively. (TIF) [file pgen.1010727.s002.tif]
