# Supplementary material for: Functional analysis of three new alpha-thalassemia deletions involving MCS-R2 reveals the presence of an additional enhancer element in the 5’ boundary region
Source: PLoS Genet. 2023 May 22;19(5):e1010727. doi: 10.1371/journal.pgen.1010727 (PMC10202303; doi:10.1371/journal.pgen.1010727)
Supplement: S1 Table — The position on the gene sequence was defined according to the Gene Bank sequence accession number NT_037887.4. (DOCX) [file pgen.1010727.s003.docx]

**S1 Table: Primers for the qRT-PCR assays. The position on the gene sequence was defined according to the Gene Bank sequence accession number NT_037887.4.**

| **Oligo forward** | | | |  | **Oligo reverse** | | | |  | **Fragments** |
| --- | --- | --- | --- | --- | --- | --- | --- | --- | --- | --- |
| **n.** | **sequence** | **Position on**  **Chr 16** | **bp** |  | **n.** | **sequence** | **Position on**  **Chr 16** | **bp** |  | **bp** |
| 1-For | ACTGGGCCTCCTTGCCTAAC | 22045-22064 | 20 |  | 1-Rev | ATCGCAGCTCACGCTGTAAAG | 22121-22101 | 21 |  | 77 |
| 2-For | GGCCTGGCTTCCTACCATAAGT | 95376-95397 | 22 |  | 2-Rev | GGCTGTTTGGAACCCACATAGA | 95445-95424 | 22 |  | 70 |
| 3-For | CTGGCCCATAAGAAGGAGGTTAA | 100478-100500 | 23 |  | 3-Rev | GGGACCTCAAGGAAGCTTATGAC | 100547-100525 | 23 |  | 70 |
| 4-For | ACGGGACAGGTTATGCAGACA | 102727-102747 | 21 |  | 4-Rev | TGCACACGTTTGAGTCTGTGTATG | 102813-102790 | 24 |  | 87 |
| 5-For | TGCTTTTACCTGTCCGTAATCAATC | 105790-105814 | 25 |  | 5-Rev | CCCATCCTCTTGCCATCAAG | 105874-105855 | 20 |  | 85 |
| 6-For | GGCCAGCAAGTCCTAATTTCTGT | 107804-107826 | 23 |  | 6-Rev | GAGCGCCCTGGTCCTTGT | 107873-107856 | 18 |  | 70 |
| 7-For | TGGTGAAGGGATGGGAGAGA | 110375-110394 | 20 |  | 7-Rev | AGTTCAAAGCAGGCCTTCCTTT | 110445-110424 | 22 |  | 71 |
| 8-For | ACCTGGAGGAAGAGGAGGTTTG | 113210-113231 | 22 |  | 8- Rev | CTGGCACCCGGGAGACTT | 113269-113252 | 18 |  | 60 |
| 9-For | CACCACATTTAAAGGGCAAGACA | 119349-119371 | 23 |  | 9-Rev | TGCTGGACACCCCATTGG | 119418-119401 | 18 |  | 70 |
| 10-For | CAGTGTGGCAGCGTTGAGA | 136053-136071 | 19 |  | 10-Rev | CCATGACAACTCATTAATCCATTCC | 136145-136121 | 25 |  | 93 |
| 11-For | GCACCGTGCTGACCTCCAAA | 163571-163590 | 20 |  | 11-Rev | CCCATCGGGCAGGAGGAA | 163636-163619 | 18 |  | 66 |
|  |  | Chr 15 |  |  |  |  | Chr 15 |  |  |  |
| B2M | TCTGCTGCGGCTCTGCTTC | 44712081-44712099 | 19 |  |  | GGTGCTAGGACATGCGAACTTAG | 44712161-44712139 | 23 |  | 80 |

B2M: β2 microglobulin
