# Supplementary material for: Functional analysis of three new alpha-thalassemia deletions involving MCS-R2 reveals the presence of an additional enhancer element in the 5’ boundary region
Source: PLoS Genet. 2023 May 22;19(5):e1010727. doi: 10.1371/journal.pgen.1010727 (PMC10202303; doi:10.1371/journal.pgen.1010727)
Supplement: S2 Table — The position of the primers on the gene sequence was defined according to the Gene Bank sequence accession number NC_000016.10. (DOCX) [file pgen.1010727.s004.docx]

**S2 Table: Primers and experimental conditions for the long-range, gap-PCR and sequencing for the definition of the breakpoints at the new deletions and for the semiquantitative analysis of the α2 globin gene cDNA and DNA. The position of the primers on the gene sequence was defined according to the Gene Bank sequence accession number NC_000016.10.**

| **Oligonucleotide** | | **Sequence 5'-3'** | **Position on chr 16** | **bp** | **pmol** | **Fragment**  **lenght (bp)** | **Annealing T** | **72°C**  **time** | **DMSO**  **%** | **Cycles**  **No.** |
| --- | --- | --- | --- | --- | --- | --- | --- | --- | --- | --- |
|  | **Long-range PCR** | | | | | | | | | |
| **(αα)ES** | Telomere-For [1] | F-TATGGATCCCTAACCCTGACCCTAACCC |  | 28 | 10 | ~ 300 | 60° | 3’ | 3.2 | 40 |
|  | 7-Rev | R-AGTTCAAAGCAGGCCTTCCTTT | 120447-12046 | 22 | 10 |  |  |  |  |  |
|  |  |  |  |  |  |  |  |  |  |  |
| **(αα)FG** | 4-For | F-ACGGGACAGGTTATGCAGACA | 112729-112749 | 21 | 10 | 2.164 | 60° | 3’ | absent | 30 |
|  | 5-Rev | R-CCCATCCTCTTGCCATCAAG | 115876-115857 | 20 | 10 |  |  |  |  |  |
|  | (αα)FG-Rev | R-TCTGTCCCTGTGTTTCCTTCAAA | 114515-114493 | 23 |  | for sequencing | | | | |
|  |  |  |  |  |  |  |  |  |  |  |
| **(αα)CT** | 4-For | F-ACGGGACAGGTTATGCAGACA | 112729-112749 | 21 | 10 | 2.661 | 60° | 3’ | absent | 30 |
|  | 7-Rev | R-AGTTCAAAGCAGGCCTTCCTTT | 120447-120426 | 22 | 10 |  |  |  |  |  |
|  | (αα)CT-For | F-GTGCCTCCCTCACCAAGGA | 113098-113116 | 19 |  | for sequencing | | | | |
|  | (αα)CT-Rev | R-CATGTGCCTTCAGTCTGTGCTT | 119493-119472 | 22 |  | for sequencing | | | | |
|  | **Gap-PCR** | | | | | | | | | |
| **(αα)ES** | Telomere-For [1] | F-TATGGATCCCTAACCCTGACCCTAACCC |  | 28 | 12 | ~ 300 | 60° | 1’ | 10 | 30 |
|  | 7-Rev | R-AGTTCAAAGCAGGCCTTCCTTT | 120447-120426 | 22 | 12 |  |  |  |  |  |
|  | Control A-For (P1) [2] | F-AGGCTGTGGGCAGAGTCAGAAGA | 175081-175103 | 23 | 1 | 714 (control) |  |  |  |  |
|  | Control B-Rev (P2) [2] | R-CAATAGCTGGAACCGGCTGGAG | 175794-175773 | 22 | 1 |  |  |  |  |  |
|  |  |  |  |  |  |  |  |  |  |  |
| **(αα)FG** | 4-For | F-ACGGGACAGGTTATGCAGACA | 112729-112749 | 21 | 10 | 803 +  1.787 (control) | 60° | 1.5’ | 10 | 30 |
|  | (αα)FG-Rev | R-TCTGTCCCTGTGTTTCCTTCAAA | 114515-114493 | 23 | 10 |  |  |  |  |  |
|  |  |  |  |  |  |  |  |  |  |  |
| **(αα)CT** | (αα)CT-For | F-GTGCCTCCCTCACCAAGGA | 113098-113116 | 19 | 10 | 1.338 | 60° | 2’ | 10 | 30 |
|  | (αα)CT-Rev | R-CATGTGCCTTCAGTCTGTGCTT | 119493-119472 | 22 | 10 |  |  |  |  |  |
|  | Control A-For (P1) [2] | F-AGGCTGTGGGCAGAGTCAGAAGA | 175081-175103 | 23 | 2 | 714 (control) |  |  |  |  |
|  | Control B-Rev (P2) [2] | R-CAATAGCTGGAACCGGCTGGAG | 175794-175773 | 22 | 2 |  |  |  |  |  |
|  | **Semiquantitative analysis** | | | | | | | | | |
| **RNA** | 12-Rev [3] | R-GGGAGGCCCATCGGGCAGGAGGAAC | 173644-173620 | 25 | 1 | for RT PCR |  |  |  |  |
|  | 13-For | F-ACTCTTCTGGTCC | 172877-172889 | 13 | 10 | 191 | 58° | 45’’ | 3.2 | 24+1 |
|  | 13-Rev (cDNA) | R-CGTGGCTCAGGTCGAAGTG | 173184-173166 | 19 | 10 |  |  |  |  |  |
|  |  |  |  |  |  |  |  |  |  |  |
| **DNA** | 14-Rev [2] (DNA) | R-GAGCAGGGGAGGGAGCGTCA | 173010-173029 | 20 | 10 | 153 | 58° | 45’’ | 3.2 | 24+1 |
|  |  |  | **MCS-R1** |  |  |  |  |  |  |  |
|  | MCR-R1-For | F-CAGTCATGTGGTCTGGTTTGTAC | 94542-94564 | 23 | 10 | 1171 | 60° | 1’ | 10 | 30 |
|  | MCR-R1-Rev | R-GAATGTGTGGGTGGGAATAACAG | 95712-95690 | 23 | 10 |  |  |  |  |  |

The underlined bases are of LNA types.

References

1. Flint J, Craddock CF, Villegas A, Bentley DP, Williams HJ, Galanello R, et al. Healing of broken human chromosomes by the addition of telomeric repeats. Am J Hum Genet. 1994;55(3):505-12. PubMed PMID: 7521575.

2. Lacerra G, Musollino G, Di Noce F, Prezioso R, Carestia C. Genotyping for known Mediterranean alpha-thalassemia point mutations using a multiplex amplification refractory mutation system. Haematologica. 2007;92(2):254-5. doi: 10.3324/haematol.10736. PubMed PMID: 17296579.

3. Cardiero G, Scarano C, Musollino G, Di Noce F, Prezioso R, Dembech S, et al. Role of nonsense-mediated decay and nonsense-associated altered splicing in the mRNA pattern of two new α-thalassemia mutants. Int J Biochem Cell Biol. 2017;91(Pt B):212-22. Epub 2017/07/22. doi: 10.1016/j.biocel.2017.07.014. PubMed PMID: 28743675.
