# Supplementary material for: Functional analysis of three new alpha-thalassemia deletions involving MCS-R2 reveals the presence of an additional enhancer element in the 5’ boundary region
Source: PLoS Genet. 2023 May 22;19(5):e1010727. doi: 10.1371/journal.pgen.1010727 (PMC10202303; doi:10.1371/journal.pgen.1010727)
Supplement: S3 Table — The sequence and the position of the oligos, respect to the MCS-R2 element or to the deletions reported in the present article, and the length of the clone are reported. Upstream of the 5’ primer was added the sequence ggtacc, recognized by the KpnI enzyme, and downstream of the 3’ primer was added gctagc, recognized by the NheI enzyme. (DOCX) [file pgen.1010727.s005.docx]

**S3 Table. Oligos designed for the directional cloning. The sequence and the position of the oligos, respect to the MCS-R2 element or to the deletions reported in the present article, and the length of the clone are reported. Upstream of the primer 5' was added the sequence *ggtacc*, recognized by the KpnI enzyme, and downstream of the primer 3' was added *gctagc*, recognized by the NheI enzyme.**

| **Clone**  **n.** | **Primer**  **Name** | **Sequence forward primers** | **Position** | **Chr16** | **Primer**  **name** | **Sequence reverse primers** | **Position** | **Chr16** | **Clone**  **lenght** |
| --- | --- | --- | --- | --- | --- | --- | --- | --- | --- |
| 1 | A | ggtaccGGAGGCTCTCAGGAACAAGA | -27/-8 MCS-R2 | 113467-113486 | B | gctagcGAATTTCTTCAGCTCCAGATGAA | +356/+334 MCS-R2 | 113849-113827 | 383 bp |
| 2 | A | ggtaccGGAGGCTCTCAGGAACAAGA | -27/-8 MCS-R2 | 113467-113486 | C | gctagcAGCCTGGCTGTGAACACTTT | +296/+277 MCS-R2 | 113789-113770 | 323 bp |
| 3-4 | D | ggtaccGCACAGGGACACAGCTGGA | -682/-664 MCS-R2 (5' del (αα)FG) | 112812-112830 | C1 | gctagcTGCCCCTGAAGCCTGGCTG | +305/+287 MCS-R2 | 113798-113780 | 987 bp |
| 5 | D | ggtaccGCACAGGGACACAGCTGGA | -682/-664 MCS-R2 (5' del (αα)FG) | 112812-112830 | E | gctagcTCTTGTTCCTGAGAGCCTCC | -27/-8 MCS-R2 | 113486-113467 | 675 bp |
| 6 | F | ggtaccCCTGCCCTGAGCAAAGACC | -332/-314 MCS-R2 | 113162-113180 | B | gctagcGAATTTCTTCAGCTCCAGATGAA | +356/+334 MCS-R2 | 113849-113827 | 688 bp |
| 7 | D | ggtaccGCACAGGGACACAGCTGGA | -682/-664 MCS-R2 (5' del (αα)FG) | 112812-112830 | B | gctagcGAATTTCTTCAGCTCCAGATGAA | +356/+334 MCS-R2 | 113849-113827 | 1038 bp |
| 8 | G | ggtaccCTGTGAAAACACTTGAGGGAGC | +89/+110 MCS-R2 (5' del (αα)CT) | 113582-113603 | B | gctagcGAATTTCTTCAGCTCCAGATGAA | +356/+334 MCS-R2 | 113849-113827 | 268 bp |
| 9 | A | ggtaccGGAGGCTCTCAGGAACAAGA | -27/-8 MCS-R2 | 113467-113486 | H | gctagcACAGACCCCCACAGCTGCA | +92/+74 MCS-R2 | 113585-113567 | 119 bp |
| 10 | D | ggtaccGCACAGGGACACAGCTGGA | -682/-664 MCS-R2 (5' del (αα)FG) | 112812-112830 | H | gctagcACAGACCCCCACAGCTGCA | +92/+74 MCS-R2 | 113585-113567 | 774 bp |
