# Supplementary material for: Functional analysis of three new alpha-thalassemia deletions involving MCS-R2 reveals the presence of an additional enhancer element in the 5’ boundary region
Source: PLoS Genet. 2023 May 22;19(5):e1010727. doi: 10.1371/journal.pgen.1010727 (PMC10202303; doi:10.1371/journal.pgen.1010727)
Supplement: S1 Methods — (PDF) [file pgen.1010727.s006.pdf]

**Functional analysis of three new α-thalassemia deletions involving MCS-R2 reveals the presence of an additional enhancer element in the 5' boundary region**

**Short title: Analysis of MCS-R2 and of its boundary**

*Serena Capasso[1#], Giovanna Cardiero[1#], Gennaro Musollino[1], Romeo Prezioso[1], Rosario Testa[2], Sabrina Dembech[3], Giulio Piluso[4], Vincenzo Nigro[4,5], F. Anna Digilio[6], & Giuseppina Lacerra[1]*.*

*[#]Serena Capasso and Giovanna Cardiero share first co-authorship*

[1]Institute of Genetics and Biophysics "Adriano Buzzati Traverso" (IGB-ABT), National Research Council (CNR), Naples, Italy,
[2]A.O.U. Policlinico Rodolico-San Marco, University of Catania, Catania, Italy,
[3]Central analysis laboratory, Azienda Ospedaliero-Universitaria, Ospedali Riuniti, Foggia, Italy,
[4]Department of Precision Medicine, University of Campania L. Vanvitelli, Naples, Italy,
[5]Telethon Institute of Genetics and Medicine (TIGEM), Pozzuoli (NA, Italy),
[6]Research Institute on Terrestrial Ecosystems (IRET-CNR), National Research Council (CNR), Naples, Italy.

*giuseppina.lacerra@igb.cnr.it

**Supplementary data**

## Materials and Methods

The extreme proximity of the SNP +14 to the 5' end of the α-globin mRNA hindered the use of the taqMan protocol because there were no conditions to draw a probe with the SNP in the middle. The digestion efficiency, performed using increasing amount (50 to 300 ng) of PCR products, each digested with 10U of NlaIV enzyme, showed 100% digestion under all conditions (S1 Fig). The digestion gives rise to a short 11 bp fragment, undetectable on gel separation because it migrates rapidly and it is lost, both in the DNA than cDNA separation. To correct the intensity of the digested shorter bands, we used as correction factor the ratio = long fragment bp/short fragment bp, respectively 153/142 and 191/180 for the DNA and cDNA digestion. The values of digested and undigested bands for each sample were then reported to 100% through a proportion.

***Firefly-Renilla Luciferase Reporter Gene Assay Constructs***

We performed the dual Firefly and Renilla Luciferase Reporter Gene Assay to evaluate the enhancer activity of different part of the MCS-R2 element[1]. The fragments of interest were amplified by PCR from human genomic DNA using the polimerases for high GC content FastStart High Fidelity (Roche) and TripleMaster (Eppendorf) according to the manifacturer's protocol and using the primers listed in Table S3, designed to perform a directional cloning. After sequencing analysis, the fragments were cloned into the pGEM-T Easy vector (Promega, Southampton, UK) in *Escherichia coli* DH5α competent cells (Promega), according to the manufacturer's instructions. White colonies were screened by colony PCR, using the same primers used for the PCR and reported above. The colonies containing the correct insert were cultured and the constructs were isolated by mini-prep protocol already reported or extracted using the NucleoSpin Plasmid Quick Pure (Macherey, Nagel)[2, 3]. The

eluted DNA was quantified, double digested with KpnI/NheI (New England BioLabs, Beverly, MA), and analyzed on agarose gel electrophoresis. To ensure the quality of the cloned fragments, constructs showing the expected pattern of digestion were sequenced, using the same primers than for the PCR protocol or the universal M13for and M13rev primers. Selected constructs were then double digested with KpnI/NheI and the insert was directly cloned into the KpnI-NheI sites of pGL3-TK Basic Vector (Promega, Madison WI) upstream of the Luciferase reporter gene. After transformation, competent *E. coli* were plated on Petri dishes and the ampicillin-resistant colonies were subjected to colony PCR, using the same primers and conditions reported above. Positive colonies were cultured. Then, the constructs were extracted with NucleoSpin Plasmid Quick Pure, quantified, and sequenced. Finally, selected colonies were cultured overnight in flasks containing LB medium supplemented with ampicillin (Sigma-Aldrich, Saint Louis, Missouri, USA). The plasmid DNA was extracted and purified using the HiSpeed Plasmid Midi Kit (Qiagen, Hilden, Germany). The eluted DNA was quantified and digested with KpnI and NheI (New England BioLabs), followed by agarose gel electrophoresis[2, 3].

***Cell Lines and Cell Culture***

The human derived bone marrow-like erythroleukemia cell line K562 were maintained in Iscove's modified Dulbecco's medium (IMDM) (Lonza Verviers, Belgium), supplemented with 10% fetal bovine serum (GIBCO, Paisley, UK), 100 U/ml penicillin/streptomycin (Lonza, Walkersville, MD, USA) and 5% CO2. For the K562 cells few minutes before the transfection $2.5x10^5$ cells were seeded in 250 μl of growth medium in the 24 multiwell tissue culture dishes[2].

***Transient Transfection and Reporter Gene Assays***

K562 cells were transfected during the log phase of their growth respectively with the PolyFects and the Attractene transfection Reagent (Qiagen, Hilden, Germany) according to the manufacturer's instructions. The K562 were transfected with 1.5 μg of each reporter gene constructs, 30 ng of the pGL3 Renilla vector and 2.25 μl of Attractene. Negative control experiments included mock cells transfections with no DNA and with an empty pGL3–TK vector. After 24 hr, transfected cells were washed with phosphate-buffered saline (PBS) (Lonza Verviers, Belgium), and lysed respectively in 500 and 100 μl of passive lysis buffer of the Dual-Luciferase Reporter Assay System (Promega, Madison, WI, USA). The lysates were transferred in 1.5 ml microcentrifuge tube, centrifuged at 13.000 rpm for 1 min at 4°C and the supernatant was collected into 1.5 ml tubes. Twenty μl from each lysate were used to measure Firefly and Renilla luciferase activities. The luminescence from the Firefly luciferase was measured with the luciferase assay Reagent II (Promega). Quenching of the Firefly luciferase and concomitant activation of Renilla luciferase was accomplished by adding Stop & Glo reagent (Promega). Luminescence was measured in a luminometer (Veritas TM Microplate Luminometer Operating Manual, Turner BioSystems, Sunnyvale, CA)[1].

The values of Firefly luciferase activity for each sample were corrected for transfection efficiency using their respective values measured for the Renilla activity. Each Luciferase/Renilla ratio was then normalized using the MCS-R2 basal activities as reference, within each experiment. The differences between the MCS-R2 fragments were calculated averaging results from three independent experiments with two/three replicates within each experiment.

Firefly luciferase was normalized against Renilla luciferase activity to account for variations in transfection efficiency and the activity associated with each fragment of the MCS-R2 expressed in relation to that measured in the MCS-R2wt construct.

## References


1. Bellini I, Pitto L, Marini MG, Porcu L, Moi P, Garritano S, et al. DeltaN133p53 expression levels in relation to haplotypes of the TP53 internal promoter region. Hum Mutat. 2010;31(4):456-65. doi: 10.1002/humu.21214. PubMed PMID: 20127977.
2. Cardiero G, Scarano C, Musollino G, Di Noce F, Prezioso R, Dembech S, et al. Role of nonsense-mediated decay and nonsense-associated altered splicing in the mRNA pattern of two new α-thalassemia mutants. Int J Biochem Cell Biol. 2017;91(Pt B):212-22. Epub 2017/07/22. doi: 10.1016/j.biocel.2017.07.014. PubMed PMID: 28743675.
3. Scialò F, Hansson BS, Giordano E, Polito CL, Digilio FA. Molecular and functional characterization of the odorant receptor2 (OR2) in the tiger mosquito Aedes albopictus. PLoS One. 2012;7(5):e36538. Epub 20120514. doi: 10.1371/journal.pone.0036538. PubMed PMID: 22606270.
